# Reductive evolution and genomic reduction in *Rhodopseudomonas*

**DOI:** 10.1128/aem.00445-25

**Published:** 2025-07-31

**Authors:** Brian M. Gallagher, Hailee M. Morrison, Arpita Bose

**Affiliations:** 1 Department of Biology, Washington University in St. Louis 7548, St. Louis, Missouri, USA; Michigan State University, East Lansing, Michigan, USA

**Keywords:** *Rhodopseudomonas*, genome reduction

## Abstract

*Rhodopseudomonas palustris* is renowned for its metabolic versatility, supported by a genome that contains pathways for numerous processes. In a recent study, Oda et al. (Appl Environ Microbiol 91:e02056-24, 2025, https://doi.org/10.1128/aem.02056-24) examine *R. palustris* DSM127, a strain with a dramatically reduced gene inventory, to study how environmental pressures have influenced gene loss. Missing more than a quarter of its genome reduces its versatility. However, it may improve its efficiency under the conditions in which it still thrives, enhancing its aptitude as a chassis for biotechnology.

## COMMENTARY

Purple non-sulfur bacteria (PNSB) possess incredibly versatile metabolisms, allowing them to perform four significant growth modes—photoautotrophy (light energy and CO_2_), photoheterotrophy (light energy and organic carbon), chemoautotrophy (chemical energy and CO_2_), and chemoheterotrophy (chemical energy and organic carbon)—in addition to specialized processes such as nitrogen fixation. This metabolic versatility enables PNSB to thrive in a wide range of environments, from wastewater ditches to marine estuaries, and in habitats that vary from fully oxygenated to completely anoxic. However, they tend to favor microaerobic and light-exposed conditions (1). PNSB are attractive candidates for many biotechnological applications. When choosing a microbial chassis for wastewater remediation, for example, it is beneficial for it to have the ability to survive under a range of conditions and degrade a diverse array of substrates, thereby giving PNSB a competitive advantage (2–4). Additionally, these organisms frequently contain complete or partial pathways for producing valuable bioproducts, such as biofuels or bioplastics (5–7).

As with most organisms, the metabolic diversity of PNSB is derived from possessing an appropriately diverse genome, containing the relevant genes necessary to create functional pathways. However, in environmental niches with limited resources, this breadth of diversity may work against the organism in question, as cellular resources are devoted to pathways that are nonessential in that context. The organism can compensate for this through natural selection, breaking these nonessential genes in a process termed “reductive evolution.” Although more commonly studied in the context of endosymbionts and extremophiles, this process can also be observed in free-living organisms (8, 9).

A more targeted approach to this concept is “genome reduction,” which focuses on the use of synthetic biology to intentionally reduce a strain’s genome to the bare minimum of genes required to sustain maximum efficient production of a target molecule (10). A significant challenge with this approach is determining which genes can be lost, a time-consuming endeavor that typically relies on a combination of computational modeling and experimental validation (10). In addition, once identified, the removal of large numbers of genes, especially in an underdeveloped genetic system, can be even more difficult. To get around this, Oda et al. co-opted the natural process of reductive evolution to assist in identifying potential target genes for removal in a recent study (11). The authors compared the PNSB strain *Rhodopseudomonas palustris* DSM127, which possesses a naturally reduced genome (approximately 4.0 Mb), with more typical *Rhodopseudomonas* strain genomes (averaging 5.5 Mb) to identify gene loss and conservation patterns. They found that DSM127 retained most growth capabilities but lacked “genes associated with high-affinity transport of nutrients, iron uptake, nitrogen metabolism, and biodegradation of aromatic compounds.” The authors propose that this reduced genome strain may be suitable for biotechnological applications that require niche environments and specialized metabolisms.

### Metabolic versatility in DSM127

*R. palustris* is a well-studied species of PNSB—its various strains are among the best characterized within the group. In addition to the typical broad PNSB metabolism, many strains of *R. palustris* possess the *pioABC* operon, which enables extracellular electron uptake from solid minerals like iron, as well as from direct sources of electrons like a poised cathode (12). This species is also the only PNSB known to encode for three types of nitrogenases, which function as metalloenzymes using molybdenum, vanadium, or iron, as opposed to most other PNSB species, which only encode for molybdenum and iron-containing nitrogenases (13, 14). Interestingly, DSM127 lacks the *pioABC* operon, as well as several genes involved in iron transport and acquisition, and retains only the molybdenum-associated nitrogenase. *R. palustris* has been experimentally shown to require a functional *pioABC* operon to perform photoferrotrophic growth, making it reasonable to predict that this strain would be unable to grow on iron (15). It is also expected that the lack of alternative nitrogenases would result in a limited capacity for nitrogen fixation, although this requires further experimental validation. Furthermore, this strain appears to encode for less than half of the reported *Rhodopseudomonas* solute binding proteins, which are associated with ABC and TRAP transporters responsible for the uptake of nutrients such as oligopeptides and aromatic compounds. The authors hypothesize that this would result in a decreased ability to compete for scarce nutrients. This ability may have been lost in response to the lack of competition in their environmental niche.

In summary, DSM127 shows a significantly reduced genome compared to the other *Rhodopseudomonas* strains examined in this study. Every COG (Cluster of Orthologous Genes) category exhibited a reduced gene inventory in DSM127 compared to its reference *Rhodopseudomonas* genomes, in many cases substantially so. This, the authors conclude, explains some of the growth defects observed in the strain on specific substrates. It is impressive, then, that DSM127 still retains so much of the metabolic versatility that is the trademark quality of *Rhodopseudomonas*.

### Metabolic regulation

Despite possessing the blueprints for building such a range of metabolic pathways, *R. palustris* must still regulate its gene expression to adapt to the environment and prevailing conditions. However, because of its versatility, the pool of possible conditions (and corresponding regulatory states) is that much larger.

*R. palustris* employs distinct sets of enzymes and biochemical pathways for aerobic and anaerobic conditions, determined most predominantly by the global regulator FixK (16). *R. palustris* FixK is homologous to the FixK2 regulator in related *Rhizobacteria* and functions as a master regulator of aerobic and anaerobic metabolism. Flipped “on” by the oxygen-sensing FixLJ, FixK transcriptionally activates dozens of genes involved in anaerobic metabolism, including the photosynthesis cluster and *pioABC* (16–18). RegRS and AadR serve similar but not identical roles, responding to other indicators of this environmental shift from oxic to anoxic conditions to regulate large swaths of the genome.

In the highlighted article, Oda et al. confirm that DSM127 preserves FixLJ-K, RegRS, and AadR, despite having lost many of the targets controlled by these regulators. Interestingly, many of the genes conspicuously absent from DSM127’s metabolic arsenal are those related to anaerobic metabolism, given the authors’ difficulty in culturing the strain aerobically. The authors report that despite retaining genes encoding for a *ccb*_3_ cytochrome *c* terminal oxidase, cytochrome *aa*_3_ oxidase, and common oxidative stress tolerance pathways, DSM127 is only able to grow microaerobically. Rather than specializing toward aerobic or anaerobic metabolism (with the corresponding atrophy of gene inventory related to the deprioritized lifestyle), we see an almost haphazard loss of metabolic capacity across both lifestyles’ inventories. Loss of *pio*-mediated photoferrotrophy and AadR-regulated anaerobic aromatic acid degradation, coupled with conservation of oxygen-tolerance genes such as catalase, might suggest a specialization of this strain toward aerobic growth. However, this is opposite to the trend observed by the authors. As they point out, the reason behind DSM127’s susceptibility to atmospheric levels of oxygen remains an open question, yet to be answered by the genome.

### Genome reduction and biotechnology

As demonstrated by Oda et al.*,* comparative genomics is a valuable tool for maximizing the ability to conclude with minimal hands-on data generation. Allowing natural selection to do the hard work of creating mutant strains means that less time is spent on cloning and trial and error at the bench. At the same time, the wealth of publicly available genomes facilitates comparisons with related strains. To answer questions about what is truly necessary for growth, it would be inefficient to individually knock out vast swathes of the genome in the laboratory, when instead we may leverage the past billion years of prokaryotic evolution and diversification to see what is maintained and what may be disposed of with or without a tradeoff.

Despite some of the baseline growth inhibition observed in DSM127, genomic reduction is not a purely deleterious process—smaller genomes pose a reduced pressure on growth in ideal conditions. Much of a given genome is, essentially, a metabolic bet-hedging strategy that ensures the organism can grow under a diverse range of restrictive conditions. Replicating DNA is a relatively slow and expensive process for a cell; for this reason, the most highly conserved genetic elements are those that are essential to growth, such as those that code for ribosomal RNA. Genetic elements that do not confer an advantage through generations are more prone to mutation or loss. Growth and competition are a brutal race for fitness, so cells will try to minimize wasting resources in unnecessary production (e.g., oxidative stress management pathways in an obligate anaerobe). Thus, cells facing the negative fitness pressure of retaining these genes under conditions where they are not needed may be more likely to eliminate these genes. By stripping away non-essential genes, such as those involved in the metabolism of an uncommon substrate, a strain sacrifices a measure of its versatility for efficiency (19).

In addition to improved efficiency in nature, genomic reduction can be a potential boon for biotechnological applications of microorganisms. Under industrial conditions, metabolic versatility may become less essential, as the feedstock would ideally be of a standard composition that can be controlled, and the efficiency of growth and reproduction becomes a more important factor. A culture may not ever encounter the conditions for anaerobic metabolism of ferrous iron or aromatics, so any gains in doubling time a strain like DSM127 would get from dropping those bet-hedging abilities may be worth the trade-off. For example, one group created a reduced *Pseudomonas putida* KT2440 genome by deleting identified genomic islands (horizontal transferable elements) that account for ~4% of the total genome (20). Compared to the base strain, the reduced strain produced 39% more polyhydroxyalkanoates (PHAs), which can be utilized as bioplastic. It exhibited improved traits, such as transformation and chromosomal integration efficiency (20). In *R. palustris* TIE-1, polyhydroxybutyrate (a type of PHA) production was found to increase with the deletion of *nifA* and *glnA*, which are required for nitrogenase functionality and glycogen synthesis, respectively (21). Most work in PNSB has thus far employed a more targeted approach to deleting specific pathways. This approach can be informed by studying how natural reductive evolution has produced similar results.

Toward this end, Oda et al. provide a first look at studying large-scale genome reduction strategies in PNSB. By examining a strain that has arisen through reductive evolution, they identify potential gene deletion candidates without the need for a full-scale experimental genome reduction project. Future work may aim to characterize how the loss of these genes in *R. palustris* affects or improves a given strain’s ability to thrive under specific growth conditions. By combining the insights gained from each such work, we step ever closer to developing a more optimal PNSB biotechnology chassis.

## EDITOR'S NOTE

**Ed. Note**: The author of this commentary served as the handling editor for the original article.
